# Evaluating the Effects of an Organic Extract from the Mediterranean Sponge *Geodia cydonium* on Human Breast Cancer Cell Lines

**DOI:** 10.3390/ijms18102112

**Published:** 2017-10-09

**Authors:** Susan Costantini, Eliana Guerriero, Roberta Teta, Francesca Capone, Alessia Caso, Angela Sorice, Giovanna Romano, Adrianna Ianora, Nadia Ruocco, Alfredo Budillon, Valeria Costantino, Maria Costantini

**Affiliations:** 1 Experimental Pharmacology Unit, Istituto Nazionale Tumori “Fondazione G. Pascale”—IRCCS, 80131 Napoli, Italy; s.costantini@istitutotumori.na.it (S.C.); e.guerriero@istitutotumori.na.it (E.G.); f.capone@istitutotumori.na.it (F.C.); a.sorice@istitutotumori.na.it (A.S.); a.budillon@istitutotumori.na.it (A.B.); 2 Department of Pharmacy, University of Naples Federico II, Via Domenico Montesano 49, 80131 Naples, Italy; roberta.teta@unina.it (R.T.); alessia.caso@unina.it (A.C.); costanti@unina.it (V.C.); 3 Department of Integrative Marine Ecology, Stazione Zoologica Anton Dohrn, Villa Comunale, 80121 Napoli, Italy; romano@szn.it (G.R.); ianora@szn.it (A.I.); 4 Department of Biology and Evolution of Marine Organisms, Stazione Zoologica Anton Dohrn, Villa Comunale, 80121 Napoli, Italy; nadia.ruocco@szn.it; 5 Department of Biology, University of Naples Federico II, Complesso Universitario di Monte Sant’Angelo, Via Cinthia, 80126 Napoli, Italy; 6 Bio-Organic Chemistry Unit, Institute of Biomolecular Chemistry-CNR, Via Campi Flegrei 34, Pozzuoli, 80078 Naples, Italy

**Keywords:** breast cancer, cytotoxicity, metabolomics, cytokines, sponges

## Abstract

Marine sponges are an excellent source of bioactive secondary metabolites for pharmacological applications. In the present study, we evaluated the chemistry, cytotoxicity and metabolomics of an organic extract from the Mediterranean marine sponge *Geodia cydonium*, collected in coastal waters of the Gulf of Naples. We identified an active fraction able to block proliferation of breast cancer cell lines MCF-7, MDA-MB231, and MDA-MB468 and to induce cellular apoptosis, whereas it was inactive on normal breast cells (MCF-10A). Metabolomic studies showed that this active fraction was able to interfere with amino acid metabolism, as well as to modulate glycolysis and glycosphingolipid metabolic pathways. In addition, the evaluation of the cytokinome profile on the polar fractions of three treated breast cancer cell lines (compared to untreated cells) demonstrated that this fraction induced a slight anti-inflammatory effect. Finally, the chemical entities present in this fraction were analyzed by liquid chromatography high resolution mass spectrometry combined with molecular networking.

## 1. Introduction

Marine sponges are conspicuous members of the marine benthos, occurring worldwide from polar and temperate to tropical seas and from the intertidal to deep-sea environments [1,2]. They have been considered a gold mine for the discovery of marine natural products during the past 50 years, with about 4851 compounds identified to date, contributing to nearly 30% of all marine natural products discovered so far [3]. Many of these products are of considerable biotechnological interest as pharmaceutical agents [4,5]. Marine sponges have given rise to two commercial anticancer compounds: (i) cytarabine or Ara-C, from the Caribbean sponge *Tethya crypta*, approved in 1969 and still used to treat acute myelocytic leukemia and non-Hodgkin’s lymphoma; and (ii) Eribulin or Halaven, a synthetic derivative based on the structure of Halichondrin B from the sponge *Halichondria okadai*, approved in 2010 for the treatment of drug refractory breast cancer. Another commercial drug obtained from sponges is vidarabine or Ara-A, a synthetic analog of spongouridine originally isolated in 1950 from the sponge *Tethya crypta*, commercialized as Vira-A^®^ and aciclovir (Zovirax^®^) for the treatment of *herpes simplex* and *herpes zoster* viruses infections [6]. Other sponge compounds are in phase I clinical trials (Hemiasterlin E7974) against cancer, or in preclinical trials against a number of other pathologies: Geodisterol sulfates (fungal infections), Plakortin (malaria), Homogenistic acid (malaria), Hymenidin (tuberculosis), Ggyrosanols (viral infections), Dysidine (diabetes), Capnellene (inflammation), Callyspongidiol (immunity), Calyculin A (nervous system), and Dysideamine (nervous system) [7]. The search for new drug candidates from sponges is therefore on the rise [8,9]. Here, we explore the biological activity of the Mediterranean sponge *Geodia cydonium* (*Porifera*, *Demospongiae*, *Astrophorida*, *Geodidae*). In a previous study, we demonstrated that a methanol extract of *G. cydonium* had an anti-inflammatory effect on the human breast cancer MCF-7 cell line by decreasing the levels of several pro-inflammatory cytokines without inducing cytotoxic effects thus indicating the potential of *G. cydonium* against human breast cancer [10].

The present study aimed to study the effects of the organic extract of *G. cydonium* on human breast cancer cell lines MCF-7, MDA-MB231 and MDA-MB468, and on normal breast cell line, MCF-10A, used as a control. The extract was fractionated by chromatography, and of the three most abundant fractions obtained, only one (fraction 3) was able to block cell proliferation of all breast cancer cell lines, with no effects on the normal breast cell line. Cellular studies were then performed to verify if this fraction induced apoptosis and/or blockage of the cell cycle. Moreover, metabolomic profiling on cells treated with fraction 3 has allowed for the identification of the metabolite pathways modulated by this fraction. Anti- or pro-inflammatory effects on three breast cancer cell lines have also been tested by cytokinome evaluation on the related cellular polar fractions. The active fraction was analyzed by liquid chromatography (LC)-high resolution mass spectrometry (HRMS) and tandem mass spectrometry (HRMS/MS) together with the molecular networking technique [11].

## 2. Results

### 2.1. Cell Proliferation

Sulforhodamine B (SRB) assay was used to identify the concentrations at which cell growth was inhibited by 50% in the breast cancer cell lines MCF-7, MDA-MB231 and MDA-MB468. None of the three fractions blocked cell proliferation in the normal cell line MCF-10A after 24 and 48 h of incubation (Figure S1). Moreover, two of the three fractions had no effects on all three human cancer cells after 24 and 48 h of treatment (Figures S2–S4). Interestingly, only one fraction blocked cell proliferation in all three human cancer cell lines.

**Figure 1:**
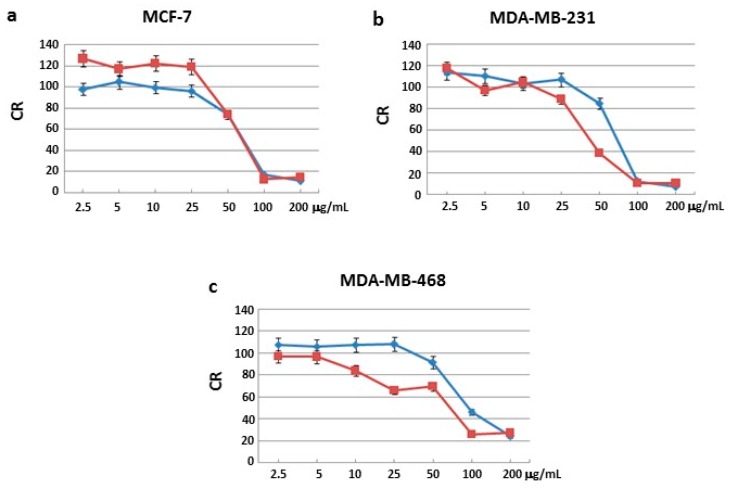
Cell proliferation. Cell viability rate (CR) related to breast cancer cells: (**a**) MCF-7; (**b**) MDA-MB231; and (**c**) MDA-MB468, after treatment with the active sponge *Geodia cydonium* sub-fraction for 24 (blue line) and 48 (red line) h.

In particular, MCF-7 cells showed a growth inhibition with the half minimal (50%) Inhibitory Concentration (IC_50_) of 72 and 67 μg/mL after 24 and 48 h of treatment, respectively (Figure 1a); MDA-MB231 cells showed a growth inhibition with an IC_50_ at 73 and 44 μg/mL after 24 and 48 h of treatment, respectively (Figure 1b); MDA-MB468 cells showed a growth inhibition with an IC_50_ at 80 and 70 μg/mL after 24 and 48 h of treatment, respectively (Figure 1c). IC_50_ values after 48 h treatment were lower compared to those obtained after 24 h.

### 2.2. Characterization by Liquid Chromatography-High Resolution Mass Spectrometry and Tandem Mass Spectrometry (LC-HRMS and LC-HRMS/MS) Combined with Bioinformatic Analyses (GNPS)

**Figure 2:**
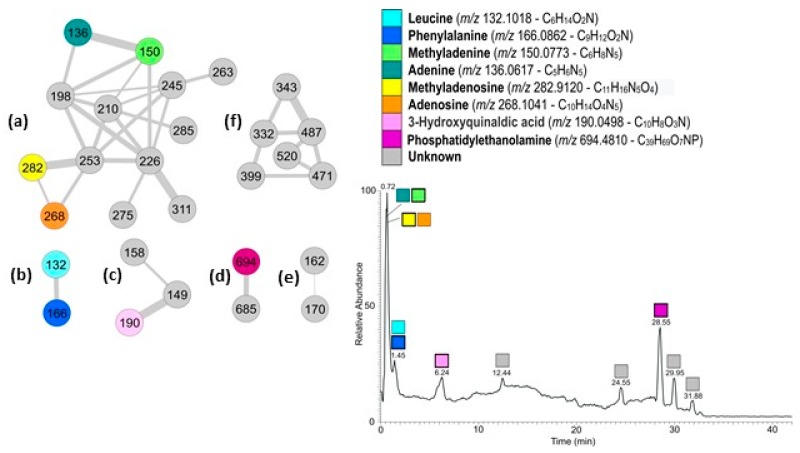
(**Left**) Two-dimensional molecular network of the active fraction from the sponge *Geodia cydonium.* In the clusters indicated with (**a**–**f**), nodes are labeled with parent *m*/*z* ratio (M+H)^+^ ions; edge thickness is related to cosine similarity score; (**Right**) Liquid chromatography-high resolution mass spectrometry (LC-HRMS) profile of the active fraction; the most abundant ions are represented by colored bold contour squares.

The active fraction was analyzed by LC-HRMS and LC-HRMS/MS (Figure 2). Data obtained were used to generate a molecular network using the Global Natural Product Social Molecular Network (GNPS). The mass spectral molecular networking resulted in a fast identification of known metabolites from natural extracts (dereplication) as well as new analogs [12]. More specifically, the spectra from one or more LC-HRMS/MS runs have been compared pairwise, and each spectrum also compared with MS/MS spectra of known natural products in GNPS libraries. The two-dimensional network obtained from the active fraction from *G. cydonium* is shown in Figure 2. A node represents a single chemical entity and its relatedness with other compounds present in the mixture is represented by an edge. The network contains six clusters ranging from two to thirteen nodes. Dereplication and search for analogs resulted in the identification of several known metabolites (Figure 2).

A more traditional approach based on the search against databases of natural and specific marine compounds (Metlin at https://metlin.scripps.edu/ and MarinLit at http://pubs.rsc.org/marinlit/) was also performed when no correspondence between molecular formula and compound name was found. Molecular networking indeed correlated compounds that share similar structural features and the dereplication workflow followed the same principle; therefore, a match within GNPS libraries does not always imply identity, but gives a significant clue on the structure of the molecule. In addition, even if libraries contain a large number of MS/MS spectra of known natural products, this number is still limited.

More specifically, cluster (a) containing thirteen nodes was the cluster containing nucleosides and nucleobases. Dereplication indicated the presence in the cluster of methyladenosine (*m*/*z* 282) and methyladenine (*m*/*z* 150). The two nodes directly connecting to these (*m*/*z* 268 and *m*/*z* 136 respectively) were also recognized by GNPS as methyladenosine and methyladenine but with a mass difference of 14 amu (atomic mass unit) with the library compounds, corresponding to a missing methyl group. Analyses of the HRMS spectra indicated the molecular formulas as C_10_H_13_O_4_N_5_ and C_5_H_5_N_5_, therefore confirming the identity of the compounds as adenosine and adenine, respectively. HRMS/MS spectra of the nucleosides revealed their characteristic fragmentation pattern: cleavage of the glycosidic bonds gave protonated bases (adenine and methyladenine) with a sugar moiety as the neutral fragment [13]. According to LC-HRMS, the relative abundance of methyladenosine is 20 times higher than adenosine, while methyladenine is double that of adenine.

Cluster (b) contained two nodes; the one with *m*/*z* 166 was dereplicated as phenylalanine (Phe), as confirmed also by the corresponding molecular formula. The second node (*m*/*z* 132) was identified as 2 amu different from pipecolinic acid (C_6_H_11_O_2_N); its molecular formula in the HRMS spectrum of C_6_H_14_O_2_N for the pseudomolecular ion peak [M+H]^+^ indicated the presence of two hydrogen atoms more than pipecolinic acid, and consequently suggested that the molecule had an open ring, and was therefore leucine (Leu). The two amino acids were found in comparable amounts in the active fraction, with Phe more abundant than Leu.

In cluster (c) the node with *m*/*z* 190.0498 has been indicated as 5-aminosalycilic acid (5-asa) in GNPS. The difference of 36 amu of the molecule with 5-asa corresponds to a C_3_ unit. Therefore, the molecule contains three more carbon atoms than 5-asa as well as three additional unsaturations. Metlin recognized the molecule as 3-hydroxyquinaldic acid (3-HQA) that perfectly matched the requests. In addition, analysis of the HRMS/MS spectrum of the molecule showed the loss of water and formic acid (HCOOH) to form the ions at *m*/*z* 172.0390 and 144.0440, but not the loss of ammonia as expected by 5-asa.

Finally, in cluster (d) there were phosphatidylethanolamine (PE) and its analogs, whereas cluster (e) and cluster (f) both comprised unknown compounds.

Due to the small amount of the networked compounds, it was not possible to establish the structure of the unknown compounds.

### 2.3. Apoptosis Increase in MCF-7, MDA-MB231 and MDA-MB468 Cells after Treatment

Based on the results obtained above, the activity of the active fraction as an apoptosis-inducer was evaluated after treatment with IC_50_ concentrations obtained after 48 h. As shown in Table 1, an increase in the number of apoptotic cells (51.2% for MCF-7, 63.1% for MDA-MB231 and 56.6% for MDA-MB468 cells) was observed compared to untreated cells used as controls.

### 2.4. RT-qPCR Analysis on MCF-7, MDA-MB231 and MDA-MB468 Cells after Treatment

**Figure 3:**
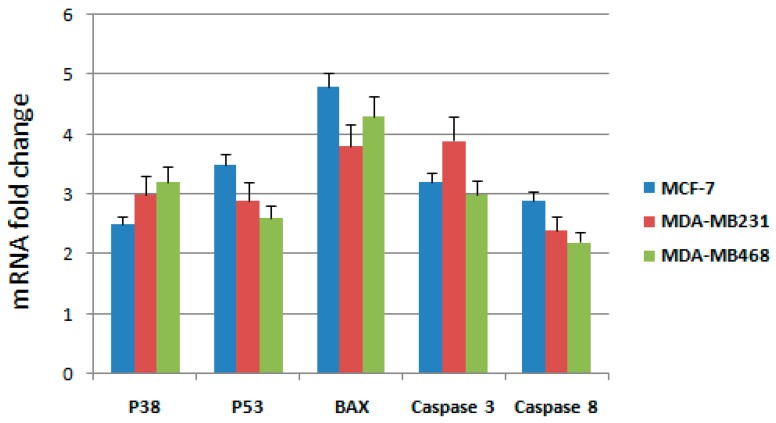
RT-qPCR analysis: mRNA fold changes were evaluated as ratios between the expression levels of five genes in three breast cancer cell lines, MCF-7, MDA-MB231 and MDA-MB468, after treatment with the active fraction compared to untreated cells.

To further elucidate the molecular mechanism through which the active fraction was able to induce apoptosis in breast cancer cells, we have examined mRNA expressions of some genes involved in the intrinsic mitochondrial pathway such as *p53*, *Bax*, *p38*, and *caspase-3*; extrinsic death receptor pathways were analyzed though the expression levels of *caspase-3* and *caspase-8* (Table 2), as already performed in our recent paper [14]. RT-qPCR was used to detect the mRNA expression after treatment with IC_50_ concentrations of the active fraction in MCF-7, MDA-MB231 and MDA-MB468 cells. Expression changes were normalized on *β-actin* mRNA expression (Figure 3).

Results showed that the mRNA expression of *p53*, *Bax*, *p38*, *caspase-3* and *caspase 8* genes increased significantly after treatment for 48 h with the active fraction in MCF-7, MDA-MB231 and MDA-MB468 cells. Taken together, these observations indicate that the active fraction induced apoptosis through both intrinsic and extrinsic pathways.

### 2.5. Analyses of the Active Fraction on the Cell Cycle of MCF-7, MDA-MB231 and MDA-MB468 Cells

Evaluation of cell cycle phases was performed on all three breast cancer lines after treatment with IC_50_ concentrations obtained after 48 h. As shown in Table 3, our results indicated that there were no visible changes for all three human breast cancer cell lines, suggesting that the active fraction had cytotoxic effects and was able to induce apoptosis but not blockage of cell cycle progression.

### 2.6. Metabolomic Profiling on Breast Cancer Cells

^1^H-NMR spectra were acquired on polar extracts from MCF-7, MDA-MB231 and MDA-MB468 cells before and after treatment with IC_50_ concentrations of the active fraction. Table 4 shows the proton resonances related to the metabolites identified in three breast cancer cells. In detail, the spectral region from 0.5 to 3 ppm contains signals from alanine, arginine, aspartate, glutamate, glutamine, isoleucine, lactate, leucine, lipids, lysine, proline, threonine and valine. The spectral region from 3 to 5.5 ppm contains mainly signals from choline, α-glucose, β-glucose, glycine, glycero-phosphocholine and phosphocholine. The 5.5–8.5 ppm region contains the resonances of histidine, phenylalanine and tyrosine.

**Figure 4:**
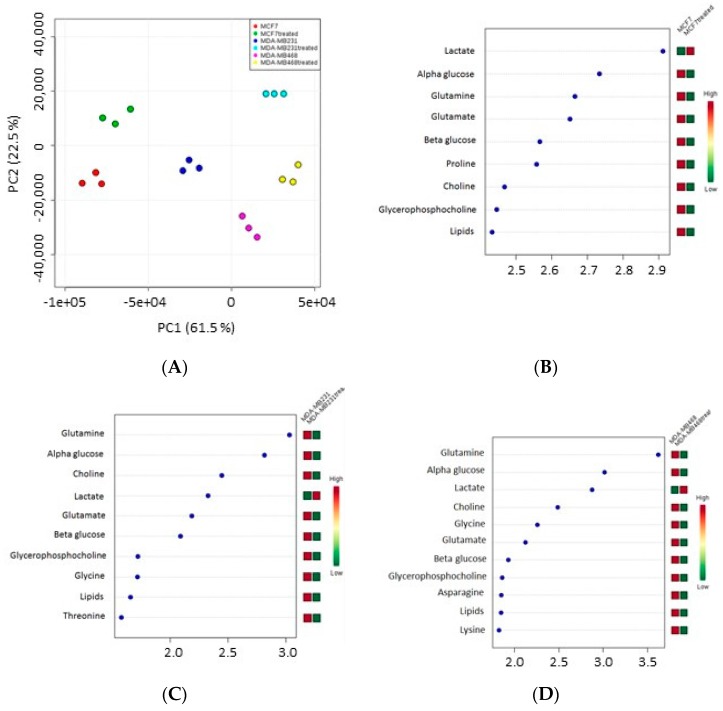
Orthogonal Projections to Latent Structures discriminant analysis (OPLS-DA) plots: (**A**) OPLS-DA and variable importance in projection (VIP) analysis where the metabolites increased or decreased in the endo-metabolome of: (**B**) MCF-7; (**C**) MDA-MB231; and (**D**) MDA-MB468 cells after treatment with the active fraction from the sponge *Geodia cydonium*, compared to untreated cells.

Orthogonal Projections to Latent Structures discriminant analysis (OPLS-DA) plot indicated that the spectra obtained for the three cell lines clustered in different groups (Figure 4A); each cell line showed statistically different proton signals and metabolites after treatment compared to controls (see variable importance in projection (VIP) score plot) (Figure 4B–D).

Data showed that the level of lactate increased after treatment in all three cell lines whereas α-glucose, β-glucose, choline, glycerophosphocholine, glutamine, glutamate and lipids decreased. The level of other metabolites also decreased: proline in MCF-7 cells, threonine in MDA-MB231, asparagine and lysine in MDA-MB468 cells, whereas glycine in both MDA-MB231 and MDA-MB468 cells.

Pathway analysis showed that the metabolites modulated by the active fraction were mainly involved in glycolysis, and lipid and amino acid metabolism (Table S1).

### 2.7. Evaluation of Cytokine Levels in Breast Cancer Cells

**Figure 5:**
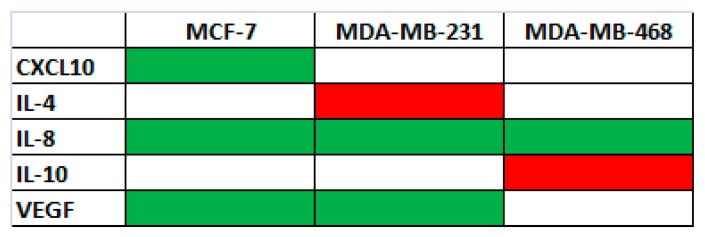
Cytokine levels in breast cancer cells. Scheme reporting the cytokines modulated by the active fraction obtained from the sponge *Geodia cydonium*. In particular, anti-inflammatory cytokines that are increased after treatment are reported in red and pro-inflammatory and pro-angiogenic cytokines that are decreased after treatment are reported in green. Blank cells indicate that the cytokines are not modulated by active fraction.

In a previous study [10], we demonstrated that a methanol extract of *G. cydonium* had an anti-inflammatory effect on the human breast cancer MCF-7 cell line by decreasing the levels of several pro-inflammatory cytokines thereby indicating the potential of *G. cydonium* against human breast cancer. In that paper [10], we evaluated the levels of the following 27 cytokines (IL-1β, IL-1ra, IL-2, IL-4, IL-5, IL-6, IL-7, CCL2, CCL11, CXCL10, CXCL8, IFN-γ, IL-9, IL-10, IL-12 (p70), IL-13, IL-15, IL-17, basic FGF, G-CSF, GM-CSF, MIP-1α, MIP-1β, PDGF-ββ, RANTES, TNF-α, and VEGF) by multiplex biometric Enzyme-Linked Immunosorbent Assay (ELISA)-based immunoassay. Hence, since the active fraction in this study derives from the same methanol extract of *G. cydonium* [10], we evaluated the same panel of 27 cytokines that comprises thirteen pro-inflammatory and anti-inflammatory interleukins, seven chemokines, five growth factors, one interferon and one tumor necrosis factor. More specifically, cytokine levels were evaluated in MCF-7, MDA-MB231 and MDA-MB468 cellular supernatants after treatment with the half minimal (50%) Inhibitory Concentration (IC_50_) concentrations of the active fraction after 48 h. Untreated cells were used as control. As shown in Figure 5, the levels of VEGF (vascular endothelial growth factor) and of two pro-inflammatory cytokines/chemokines, IL-8 (CXCL8-CXC chemokine 8) and CXCL10 (chemokine interferon-γ inducible protein 10 kDa), and two anti-inflammatory interleukins, IL-4 and IL-10, changed after treatment.

In particular: (i) in MCF-7, MDA-MB231 and MDA-MB468 cells, the levels of IL-8 decreased; (ii) in MCF-7 and MDA-MB231 cells, the levels of VEGF decreased; (iii) only in MCF-7 the level of CXCL10 decreased; (iv) only in MDA-MB231 cells the level of IL-4 increased; and (v) only in MDA-MB468 cells the level of IL-10 increased.

## 3. Discussion

Whereas several studies have focused on marine sponges with the aim of identifying natural compounds with pharmacological activity, few studies have been performed on the marine sponge *G. cydonium*. Very old studies identified steroidal ketones from this species [15].

Our data revealed that an active fraction obtained from *G. cydonium* induced apoptosis, differentially increasing the number of apoptotic cells in the three different breast cancer cell lines. These differences in the percentage of apoptotic cells for the three breast cancer cell lines may be attributed to their different nature. In fact, MCF-7 cells are estrogen-receptor-positive, whereas MDA-MB231 and MDA-MB468 cells are estrogen-receptor-negative. In addition, the triple-negative human breast cancer cells, MDA-MB231 and MDA-MB468, are characterized by a more malignant phenotype [16,17] because they constitutively express mutations of the tumor protein p53, whereas MCF-7 cells have the wild-type p53.

An important outcome from our studies was that metabolomic profiling revealed different metabolites in the three breast cancer cells MCF-7, MDA-MB231 and MDA-MB468. Pathway analysis showed that the metabolites modulated by the active fraction were mainly involved in glycolysis, and lipid and amino acid metabolism. It is well known that most tumors are highly dependent on glucose to support bioenergetic and macromolecular synthesis. In fact, tumor cells have a higher rate of glucose consumption through a different glycolysis pathway compared to normal cells, in which there is the conversion of pyruvate to lactate [18]. Thus, glucose consumption is an important step for cancer cells because the deprivation of glucose can induce their death [19,20]. Our data show that the active sponge fraction produced a decrease in glucose levels and an increase in lactate deriving from the unbalanced conversion of glucose to lactate in all three breast cancer cells. Considering that glutamine, glycine and glutamate originate from glucose metabolism via glycolysis intermediates, a decrease in their levels may be correlated to a decrease in glucose levels.

We also observed a decrease in choline levels after treatment with sponge extract. Choline is necessary for normal membrane formation, and its abnormal metabolism is considered a metabolic hallmark associated to oncogenesis and tumor progression [21]. Its decrease in the three breast cancer cell lines after treatment suggests that our active sponge fraction may be able to block cancer progression.

Different cytokines were also decreased by the sponge extract, including VEGF, CXCL10, and IL-8. VEGF is a powerful angiogenic factor and has been shown to have a role in tumor angiogenesis. In particular, it has a key role in the development, progression and metastasis of different types of cancers [22]. CXCL10 is a pro-inflammatory chemokine which modulates innate and adaptive responses of the immune system and/or regulation of cell growth and angiogenesis. Since its induction is associated with several human diseases including cancer, it is currently in use for cancer target therapy [23]. IL-8 is a pro-inflammatory chemokine, and its increased expression in different cell types such as endothelial cells, infiltrating neutrophils, cancer cells, and tumor-associated macrophages suggests that it may be an important regulatory factor within the tumor microenvironment which promotes proliferation, survival, and migration of endothelial, cancer cells, and infiltrating leukocites [24].

We also observed a slight increase in levels of IL-4 and IL-10. These are cytokines with anti-inflammatory activity and properties involved in antitumor response [25]. Our data therefore corroborate previous findings showing that a methanolic extract from the same sponge decreased pro-inflammatory cytokine levels in the human breast cancer cell line MCF-7 [10].

The main constituents of the active sponge fraction were identified as: (i) methyladenine, methyladenosine, adenine and adenosine in cluster (a); (ii) leucine and phenylalanine in cluster (b); (iii) 3-HQA in cluster (c); and (iv) phosphatidylethanolamine in cluster (d). The metabolites identified in cluster (a) are nucleosides (methyladenine and methyladenosine) and nucleotides (adenine and adenosine) that are vital components of all living cells involved in several key biological processes (nucleic acid synthesis). Marine nucleosides have already been shown to have antiviral, anticancer, vasodilator, muscle relaxant, and hypertensive activities and are currently providing new lead compounds for drug design [26]. The amino acids (leucine and phenylalanine) in cluster (b) are known to occur both in the free-state and as basic units of proteins and other metabolites (peptides) in marine organisms such as sponges and associated bacteria. Marine amino acid derivatives and peptides have been shown to possess interesting biological properties; in particular, they have attracted much attention due to their high specificity against cancer cell lines [27]. 3-HQA in cluster (c) is found in several bisintercalator natural products of marine origin (such as thiocoraline, triostin, SW-163 and echinomycin/quinomycin) [28], as one of the two key chromophores (together with quinoxalinic acid) for binding to duplex DNA by insertion between the bases to allow proper placement of the peptidic core into the DNA minor groove [29]. Phosphatidylethanolamine (PE) in cluster (d) belongs to the family of phospholipids and glycolipids that are the main constituents of sponge cell membranes [30,31], and have been shown to possess immune-modulating and antitumor activity [32,33].

In conclusion, the presence of these compounds in our extract suggests that they could be responsible for the observed changes in the metabolic profiling and cytokine secretion observed in the three cancer cell lines. Our results represent an interesting finding considering that triple negative breast cancer cells (MDA-MB231 and MDA-MB468) represent 15% of all breast cancer cases and are associated with a high malignancy and low chances of survival compared to estrogen-positive cells. Further studies are necessary to understand the mechanism of action of the single compounds present in the active fraction from *G. cydonium* and to evaluate their efficacy compared to other drugs used for the treatment of breast cancer cells. It would also be interesting to evaluate the possibility of a synergistic effect of a pool of these compounds to improve the effect of chemotherapy treatments against the proliferation of breast cancer cells, as already shown for hepatocellular carcinoma [34].

## 4. Materials and Methods

### 4.1. Collection, Extraction, and Separation

Several samples of the sponge *Geodia cydonium* (Order Tetractinellida, Family Geodiidae) were collected on July 2015 at 20 m depth by scuba diving in the “Parco Sommerso di Baia” (Gulf of Naples, Italy). After collection, the samples were immediately frozen and stored at −20 °C until extraction. The sponges (509 g wet weight) were homogenized and extracted with MeOH (2 × 2 L), MeOH and CHCl_3_ in different ratios (2:1, 1:1, 1:2) and then with CHCl_3_ (2 × 2 L) [35]. The MeOH extracts were partitioned between H_2_O and n-BuOH [36]; the BuOH layer was combined with the CHCl_3_ extracts and concentrated in vacuo. The resulting organic extract (3.9 g) was chromatographed by Droplet CounterCurrent Chromatography (DCCC) using CHCl_3_/CH_3_OH/H_2_O (7:13:8) in the ascending mode; 6 mL fractions were collected and combined in ten fractions based on their similar Thin Layer Chromatography (TLC) retention times. The most abundant fractions were tested to evaluate their anti-proliferative activity on three human breast cancer cell lines, MDA-MB231, MDA-MB468 and MCF-7, and the normal human breast epithelial cell line MCF-10A.

### 4.2. LC-HRMS and LC-HRMS/MS and Molecular Networking Analyses

Experiments were performed using a Thermo LTQ Orbitrap XL high-resolution ElectroSpray Ionization (ESI) mass spectrometer coupled to an Agilent model 1100 LC system, which included a solvent reservoir, in-line degasser, binary pump, and refrigerated autosampler. A 5 μm Kinetex C18 column (50 × 2.1 mm), maintained at 25 °C, was operated using a gradient elution of H_2_O and MeOH running at 200 μL/min. The gradient program was as follows: 10% MeOH for 5 min, 10–100% MeOH over 25 min, 100% MeOH for 13 min. All the mass spectra were recorded in the positive-ion mode. MS parameters were a spray voltage of 4.8 kV, a capillary temperature of 285 °C, a sheath gas rate of 32 units N_2_ (ca. 320 mL/min), and an auxiliary gas rate of 15 units N_2_ (ca. 150 mL/min). Data were collected in the data-dependent acquisition (DDA) mode, in which the first and second most intense ions of a full-scan mass spectrum were subjected to tandem mass spectrometry (MS/MS) analysis. MS/MS scans were obtained for selected ions with collision induced dissociation (CID) fragmentation, isolation width 2.0, normalized collision energy 36, Activation Q 0.250, and activation time 30 ms. Mass data were analyzed using the Thermo Xcalibur software (ThermoFisher Scientific, Waltham, MA, USA).

A molecular network was created using the online workflow at GNPS. The data was then clustered with MS-Cluster with a parent mass tolerance of 2.0 Da and a MS/MS fragment ion tolerance of 0.5 Da to create consensus spectra. Further, consensus spectra that contained less than 2 spectra were discarded. A network was then created where edges were filtered to have a cosine score above 0.6 and more than 6 matched peaks. Further edges between two nodes were kept in the network if and only if each of the nodes appeared in each other’s respective top 10 most similar nodes. For dereplication purposes the spectra in the network were then searched against GNPS’ spectral libraries. All matches kept between network spectra and library spectra were required to have a score above 0.6 and at least 6 matched peaks. Analog search was enabled against the library with a maximum mass shift of 100.0 Da. The data were then imported into Cytoscape 3.2.1 (Available online: http://www.cytoscape.org/) and displayed as a network of nodes and edges. The network was organized with the preferred layout plug-in.

### 4.3. Cell Culture

Three human breast cancer cell lines, MDA-MB231, MDA-MB468 and MCF-7, all derived from adenocarcinoma metastasis and on normal human breast epithelial cells MCF-10A were used. In particular, MCF-7 and MCF-10A cells were expanded at 37 °C in a humidified atmosphere of 5% CO_2_ in culture medium DMEM (Dulbecco’s Modified Eagle’s Medium, Lonza), whereas MDA-MB-231 and MDA-MB-468 in RPMI 1640 (Lonza, Munchensteinerstrasse, Basel, Switzerland), supplemented with fetal bovine serum (FBS) (Invitrogen, Camarillo, CA, USA) at 10%, Penicillin/Streptomycin 100x (Euroclone, Devon, UK), Glutamax 100x (Invitrogen) non-essential amino acids 100x (Invitrogen). Moreover, in the case of MCF-10A the DMEM was supplemented also with human insulin 10 μg/mL (Life Technologies Corporation, Carlsbad, CA, USA), human epidermal growth factor 20 ng/mL (Life Technologies), and hydrocortisone 0.5 μg/mL (Sigma-Aldrich, St. Louis, MO, USA) according to the procedure reported in Rothwell et al. (2014), while for MDA-MB468 the medium was implanted with Ham’s F-12 medium (1:1 mixture). Phosphate buffer (PBS phosphate buffered saline Ca^2+^ and Mg^2+^ free) and trypsin (Ca^2+^ and Mg^2+^ free) were supplied by Euroclone. Finally, the cells were kept in an incubator at a humidified atmosphere of 95% air and 5% CO_2_ at 37 °C.

### 4.4. Cell Treatment and Cell Proliferation Assay

Cell proliferation of cancer cells was assessed in the presence and absence of the methanol extract from *G. cydonium* by colorimetric assay with sulforhodamine B (SRB, Sigma-Aldrich). This extract was first dissolved in dimethyl sulfoxide (DMSO 100 mM, Sigma-Aldrich) at concentrations <0.1%, so as not to induce toxic effects on cells. Thus, a stock solution (100 mg/mL) and its serial dilutions had a final concentration of DMSO of 0.05%.

Cancer cells were plated in 96 well tissue culture plates at a concentration of 15 × 10^3^ cells per well and allowed to attach for 24 h. Cells were then treated with different concentrations of the methanol extract (2.5, 5, 10, 25, 50, 100, and 200 μg/mL) and incubated for 24 and 48 h. These concentrations were selected on the basis of our recent paper [10]. After 48 h of treatment, cells were fixed with trichloroacetic acid (Sigma-Aldrich) for 1 h at 4 °C. Subsequently they were stained for 30 min with 0.4% (wt/vol) sulforhodamine B (SRB, Sigma-Aldrich) dissolved in 1% acetic acid. The number of viable cells was directly proportional to the amount of protein bound-dye, which was then solubilized with 10 mM Tris base solution (pH 10.5) and measured at 540 nm using the ELISA fluorometric assay (Bio-Rad, Hercules, CA, USA; Microplate Reader). All experiments were performed in duplicate and repeated three times. The IC_50_ was assessed from the dose-response curves.

### 4.5. Apoptosis Evaluation

After counting, 3 × 10^5^ cells were harvested and washed twice with ice-cold PBS. Cells were labeled with an Annexin V and Dead Cell Assay kit according to the manufacturer’s instructions (Merck Millipore, Darmstadt, Germany). The kit detects the externalization of phosphatidylserine (PS) in apoptotic cells using fluorescently-labeled Annexin V in combination with the dead cell marker 7-aminoactinomycin D (7-AAD). We identified four populations of cells: (1) viable cells that did not undergo detectable apoptosis: Annexin V (−) and dead cell marker (−); (2) early apoptotic cells: Annexin V (+) and dead cell marker (−); (3) late apoptotic cells: Annexin V (+) and dead cell marker (+); and (4) cells that died via non-apoptotic pathways: Annexin V (−) and dead cell marker (+). Cells were counted using the Muse™ Cell Analyzer (Merck Millipore) and analyzed using software provided by Merck Millipore.

### 4.6. Cell Cycle Assay

In total, 1 × 10^6^ cells were counted for the Muse™ Cell Cycle Assay that consisted in the use of the nuclear DNA intercalating stain RNAse A and propidium iodide (PI) in a proprietary formulation. The latter was used to discriminate cells in different phases of the cell cycle, based on differential DNA content in the presence of RNAse to increase the specificity of DNA staining. After treatment with the active *G. cydonium* fraction, cells were washed with phosphate buffered saline (PBS) and centrifuged. The supernatant was removed and 1 mL of ice cold 70% ethanol was added to the re-suspended cell pellet. Samples were capped and frozen at −20 °C for at least 3 h prior to staining. Ethanol-fixed cells were washed with PBS and incubated with 200 μL of Muse™ Cell Cycle Reagent for 30 min at room temperature, in the dark. After staining, cells were processed for cell cycle analysis.

### 4.7. RNA Extraction and Real Time qPCR (RT-qPCR)

RNA isolation and cDNA preparation were performed as previously described [10]. The reverse-transcribed products were used to perform a qPCR in order to evaluate the expression level of transcripts of selected genes. Sequences for mRNAs from the nucleotide data bank (National Center for Biotechnology Information, Bethesda, MD, USA) were used to design primer pairs for RT-qPCR (Primer Express, Applied Biosystems, Foster City, CA, USA). Oligonucleotides were obtained from Sigma-Aldrich. The primer sequences are provided in Table 2.

Starting with 2 μg of total RNA, we prepared 20-fold dilution of the resulting cDNA to achieve the concentration equivalent to 100 ng of RNA (Life Technologies–Invitrogen), according to the manufacturer’s instructions. Ten nanograms of cDNA were amplified in a total volume of 25 μL containing 1X SYBR Green PCR Master Mix (Applied Biosystems) and 300 nM of forward and reverse primers. The thermal profile conditions were as follows: 5 min of denaturation at 95 °C followed by 44 cycles at 95 °C for 30 s and 60 °C for 1 min. We have added one cycle for melting curve analysis at 95 °C for 15 s, 60 °C for 15 s and 95 °C for 15 s to verify the presence of a single product. Melting-curve analysis was carried out after amplification to verify the validity of the amplicon. Each assay included a no-template control for each primer pair. To capture intra-assay variability, all RT-qPCR reactions were carried out in triplicate. For all RT-qPCR experiments, the data from each cDNA sample were normalized using β-actin mRNA as endogenous level [10]. Sample Δ*C*_t_ values were calculated as the difference between the means of gene markers *C*_t_ and housekeeping assay *C*_t_ from the same sample. The 2x-fold expression level was chosen as the threshold for significance of target genes. Statistical analyses (paired Student’s *t*) were performed using Prism software (Graphpad Software, La Jolla, CA, USA).

### 4.8. Extraction of the Polar Fractions in Untreated and Treated Cancer Cells

All cancer cell lines were plated in cell culture flasks (~2 × 10^6^ cells/flask) and treated with the active fraction at the IC_50_ concentration obtained after 48 h treatment. After incubation time (48 h), cellular supernatants were collected and stored at −80 °C for further investigation. Cell pellets obtained by trypsin digestion were washed twice in Phosphate buffered saline and deuterated water (PBS–D_2_O) and refrigerated at −80 °C. Subsequently they were re-suspended in 170 μL of H_2_O and 700 μL of methanol and were sonicated for 30 s. Then, 350 μL of chloroform was added and cell pellets were shaken on ice in an orbital shaker for 10 min. H_2_O/chloroform (350 μL, 1:1, *v*/*v*) was added to each cell suspension and centrifuged at 10,000 rpm for 10 min at 4 °C. Thereafter, the aqueous (polar) and lipophilic (apolar) phases were collected separately and evaporated by SpeedVac system.

### 4.9. ^1^H-NMR Metabolomic Analysis of the Cellular Polar Fractions

A 600-MHz Bruker Avance DRX spectrometer with a cryoprobe was used to acquire ^1^H spectra on the cellular polar fractions. They were dissolved in 630 μL of PBS–D_2_O with the pH adjusted to 7.20, and 70 μL of sodium salt of 3-(trimethylsilyl)-1-propanesulfonic acid (1% in D_2_O) used as the internal standard.

All ^1^H-NMR spectra were acquired at 300 K with the excitation sculpting pulse sequence to suppress water resonance. A double-pulsed field gradient echo was used, with a soft square pulse of 4 ms at the water resonance frequency and with gradient pulses of 1 ms duration, adding 128 transients of 64,000 complex points, with an acquisition time of 4 s/transient. Time domain data were all zero-filled to 256,000 complex points and an exponential amplification of 0.6 Hz was applied prior to Fourier transformation.

### 4.10. Statistical and Pathway Analysis

The spectral 0.50–8.60 ppm region of ^1^H-NMR spectra was integrated in buckets of 0.04 ppm by the AMIX package (Bruker, Biospin GmbH, Rheinstetten, Germany). The water resonance region (4.5–5.2 ppm) was excluded during the analysis and the bucketed region was normalized to the total spectrum area using Pareto scaling. Orthogonal Projections to Latent Structures discriminant analysis (OPLS-DA) was used to compare the spectra obtained on the polar phases from three breast cancer cell lines before and after treatment because OPLS-DA can more effectively cope with chemical shift variation in full-resolution ^1^H-NMR datasets [37] without requiring binning or alignment steps. Pathway analysis on the metabolites that were modulated after treatment was performed using Metabo Analyst tool [38].

### 4.11. Bio-Plex Assay

Several cytokines, chemokines, and growth factor levels were evaluated concurrently with the Bio-Plex assay that containing dyed microspheres conjugated with a monoclonal antibody highly specific for a target protein. The method was carried out according to the manufacturer’s instructions (Bio-Plex Bio-Rad) to assess the cytokines levels. The Bio-Plex Pro Human Cytokine 27-Plex Immunoassay has been used on supernatants of the three lines of human breast cancer after treatment with sponge extract concentrations. This panel consists of: IL-1β, IL-1ra, IL-2, IL-4, IL-5, IL-6, IL-7, CCL2, CCL11, CXCL10, CXCL8, IFN-γ, IL-9, IL-10, IL-12 (p70), IL-13, IL-15, IL-17, basic FGF, G-CSF, GM-CSF, MIP-1α, MIP-1β, PDGF-ββ, RANTES, TNF-α, and VEGF. Protein levels were determined using a Bio-Plex array reader (Luminex, Austin, TX, USA) that quantifies multiplex immunoassays in a 96-well format with very small fluid volumes. The analyte level was calculated using a standard curve, with software provided by the manufacturer (Bio-Plex Manager Software, Austin, TX, USA). A Bio-Plex array reader (Luminex, Austin, TX, USA) that quantifies multiplex immunoassays in a 96-well format with very small fluid volumes, has been used for protein level determination. The levels of the analytes were calculated using a standard curve, with the Bio-Plex Manager Software provided by the manufacturer.

## Figures and Tables

**Table 1:** Apoptosis. Percentage of live, apoptotic and dead cells expressed as mean ± standard deviation by the Muse Annexin V and Dead Cell assay in MCF-7, MDA-MB231 and MDA-MB468 cells after treatment with IC_50_ concentrations obtained after 48 h. Untreated cells were used as control.

Cells	Live (%)	Apoptosis (%)	Dead (%)
MCF-7 untreated	94.8 ± 2.4	3.8 ± 2.6	1.4 ± 0.8
MCF-7 treated	47.8 ± 1.8	51.2 ± 1.1	0.9 ± 0.4
MDA-MB231 untreated	97.7 ± 3.2	2.1 ± 2.3	0
MDA-MB231 treated	36.4 ± 3.9	63.1 ± 2.5	0
MDA-MB468 untreated	96.3 ± 2.4	3.5 ± 2.3	0.30 ± 0.05
MDA-MB468 treated	38.7 ± 3.2	56.6 ± 3.1	3.9 ± 0.9

**Table 2:** Primer sequences of the genes used in this study.

Gene Name	Primer Sequence (5′→3′)
*P38MAPK*	GCC CAA GCC CTT GCA CAT (18)
	TGG TGG CAC AAA GCT GAT GAC (21)
*P53*	CTG GCC CCT GTC ATC TTC TG (20)
	CCG TCA TGT GCT GTG ACT GC (20)
*Bax*	GGA CGA ACT GGA CAG TAA CAT GG (23)
	GCA AAG TAG AAA AGG GCG ACA AC (23)
*caspase-3*	CAGTGGAGGCCGACTTCTTG (20)
	TGGCACAAAGCGACTGGAT (19)
*caspase-8*	GGATGGCCACTGTGAATAACTG (22)
	TCGAGGACATCGCTCTCTCA (20)
*β-Actin*	TCT GGC ACC ACA CCT TCT ACA ATG (24)
	AGC ACA GCC TGG ATA GCA ACG (21)

**Table 3:** Cell percentages in the cell cycle phases (gap 0 (G0), gap1 (G1), synthesis (S), gap2 (G2) and mitosis (M) phases) expressed as mean ± standard deviation by the Muse Cell cycle assay in MCF-7, MDA-MB231 and MDA-MB468 cells after treatment with IC_50_ concentrations obtained after 48 h. Untreated cells were used as control.

Cells	G0/G1	S	M
MCF7 untreated	51.7 ± 2.3	17.3 ± 2.7	30.7 ± 1.7
MCF7 treated	45.3 ± 2.1	14.2 ± 3.4	31.2 ± 2.3
MDAMB231 untreated	59.1 ± 3.2	16.9 ± 3.9	21.8 ± 2.4
MDAMB231 treated	63.7 ± 1.4	15.2 ± 4.3	18.8 ± 2.3
MDAMB468 untreated	47.1 ± 3.9	16.9 ± 4.5	33.7 ± 1.8
MDAMB468 treated	39.2 ± 4.9	14.2 ± 2.9	39.1 ± 2.2

**Table 4:** List of ^1^H chemical shift (ppm) of metabolites found in three breast cancer cells.

Metabolites	Group	Chemical Shift	Metabolites	Group	Chemical Shift
Lipids	C_18_H_3_	0.6–0.68	α-Glucose	C4H	3.42
Lipids	C_26_H_3_	0.87–0.89	β-Glucose	C5H	3.47
Leucine	δCH_3_	0.96	β-Glucose	C3H	3.48
Valine	γCH_3_	0.97	α-Glucose	C2H	3.54
Valine	βCH_3_	1.04	Glycine	CH_2_	3.56
Threonine	γCH_3_	1.20	Phosphocholine	NCH_2_	3.6
Isoleucine	γCH_2_u	1.24	Valine	αCH	3.63
Threonine	γCH_3_	1.32	Glycero-phosphocholine	NCH_2_	3.68
Lactate	βCH_3_	1.34	α-Glucose	C3H	3.72
Isoleucine	γCH_2_u	1.46	Alanine	αCH	3.75
Alanine	βCH_3_	1.48	Glutamine	αCH	3.76
Lipids	COCH_2_CH_2_	1.59–1.61	Glutamate	αCH	3.77
Leucine	βCH_2_	1.72	α-Glucose	C6H	3.78
Lysine	δCH_2_	1.72	α-Glucose	C5H	3.84
Lysine	βCH_2_	1.90	β-Glucose	C6H	3.90
Arginine	βCH_2_	1.91	Phenylalanine	αCH	4.02
Glutamate	βCH	2.04	Choline	αCH_2_	4.07
Proline	βCH_2_	2.06	Lactate	αCH	4.11
Glutathione	βCH_2_	2.14	Proline	αCH	4.12
Glutamine	βCH_2_	2.15	Phosphocholine	OCH_2_	4.16
Valine	βCH	2.28	Threonine	βCH	4.26
Glutamate	γCH_2_	2.34	Glycero-phosphocholine	OCH_2_	4.32
Proline	βCH_2_d	2.35	β-Glucose	C1H	5.2
Glutamine	γCH_2_	2.43	α-Glucose	C1H	5.24
Aspartate	β′CH_2_	2.79	Histidine	C4H	6.91
Lysine	εCH_2_	3.03	Histidine	C4H′	6.99
Phosphocholine	N(CH_3_)_3_	3.18	Tyrosine	C2,6H	7.15–7.2
Choline	N(CH_3_)_3_	3.19	Tyrosine	C2,6H′	7.18
Arginine	δCH_2_	3.22	Phenylalanine	C4H	7.33
Glycero-phosphocholine	N(CH_3_)_3_	3.22	Phenylalanine	C2,6H	7.39
β-Glucose	C2H	3.26	Phenylalanine	C3,5H	7.43
Proline	CH_2_u	3.34	Histidine	C2H	7.78

## Supplementary Materials

Supplementary materials can be found at www.mdpi.com/1422-0067/18/10/2112/s1.
