# Supplementary material for: Evaluating the Effects of an Organic Extract from the Mediterranean Sponge Geodia cydonium on Human Breast Cancer Cell Lines
Source: Int J Mol Sci. 2017 Oct 9;18(10):2112. doi: 10.3390/ijms18102112 (PMC5666794; doi:10.3390/ijms18102112)
Supplement: Supplementary file 1 [file ijms-18-02112-s001.pdf]

Evaluating the effects of an organic extract from the Mediterranean sponge Geodya cydonium on human breast cancer cell lines

Susan Costantini[1], Eliana Guerriero[1], Roberta Teta[2], Francesca Capone[1], Alessia Caso[2], Angela Sorice[1], Giovanna Romano[3], Adrianna Ianora[3], Nadia Ruocco[4,5,6], Alfredo Budillon[1], Valeria Costantino[2], Maria Costantini[4]

[1]SC Farmacologia Sperimentale - Istituto Nazionale per lo Studio e la cura dei Tumori "Fondazione G. Pascale", IRCCS, 80131 Napoli, Italy

[2] Department of Pharmacy, University of Naples Federico II, Via Domenico Montesano, 49 80131 Naples, Italy

[3]Department of Integrative Marine Ecology, Stazione Zoologica Anton Dohrn, Villa Comunale, 80121 Napoli, Italy

[4]Department of Biology and Evolution of Marine Organisms, Stazione Zoologica Anton Dohrn, Villa Comunale, 80121 Napoli, Italy

[5]Department of Biology, University of Naples Federico II, Complesso Universitario di Monte Sant'Angelo, Via Cinthia, 80126, Napoli, Italy

[6]Bio-Organic Chemistry Unit, Institute of Biomolecular Chemistry-CNR, Via Campi Flegrei 34, Pozzuoli, Naples 80078, Italy

Table S1. List of metabolic pathways in which the significant metabolites in the polar phases of three breast cancer cell lines are involved

Pathway	Molecules
Common between three cell lines	
Glycolysis and Gluconeogenesis	Lactate, alpha-glucose, beta-glucose
Glycerophospholipidmetabolism	Choline and glycerophosphocholine
Glutamine and glutamatemetabolism	glutamine and glutamate
Specific in MCF-7	
Aminoacyl-tRNAbiosynthesis	glutamine and proline
Specific in MDA-MB231	
Glycine, Serine, Threoninemetabolism	Choline, Glycine, Threonine
Nitrogenmetabolism	Glutamine, Glycine
Aminoacyl-tRNAbiosynthesis	Glutamine, Glycine, Threonine
Specific in MDA-MB468	
Aminoacyl-tRNAbiosynthesis	Glutamine, Glycine, Lysine, Asparagine
Nitrogenmetabolism	Glutamine, Glycine, Asparagine
Cyanoamico acid metabolism	Glutamine, Glycine, Asparagine
Alanine, aspartate and glutamatemetabolism	Glutamine, Asparagine
Lysinedegradation	Glycine, Lysine

Fig S1

a

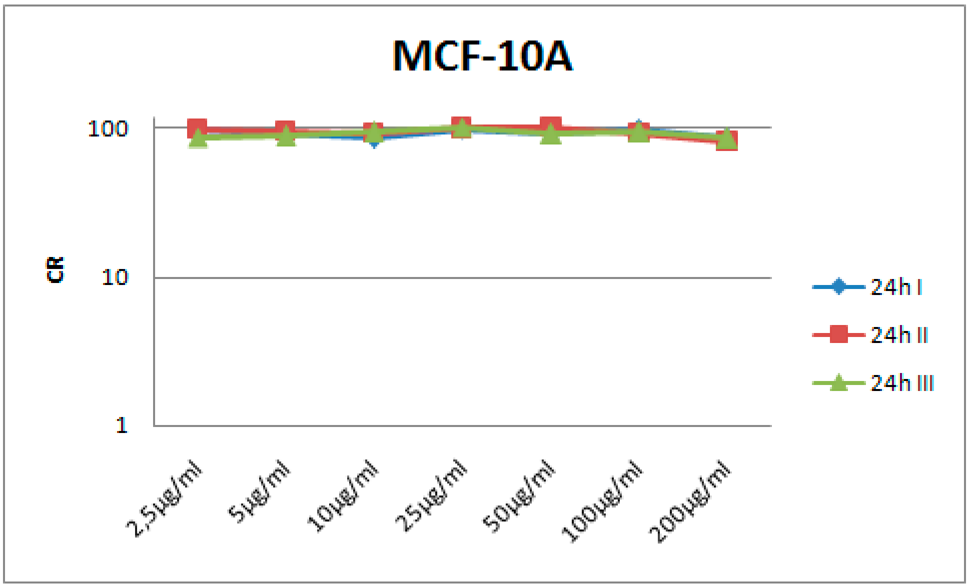

b

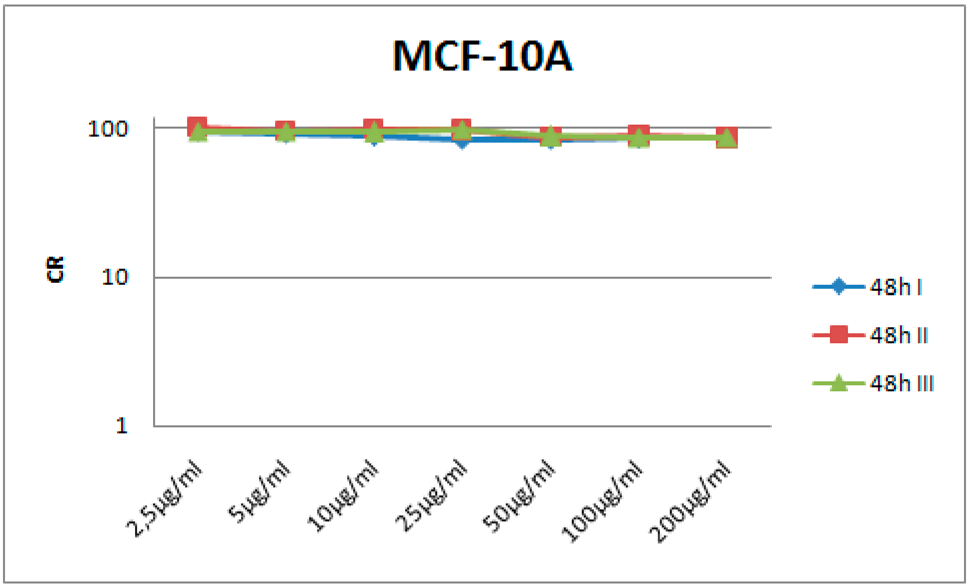

Fig S1. Cell viability (CR) related to normal breast cells, MCF-10A, after the treatment with three sponge sub-fractions named 1, 2 and 3 for (a) 24 and (b) 48 h.

Fig S2

a

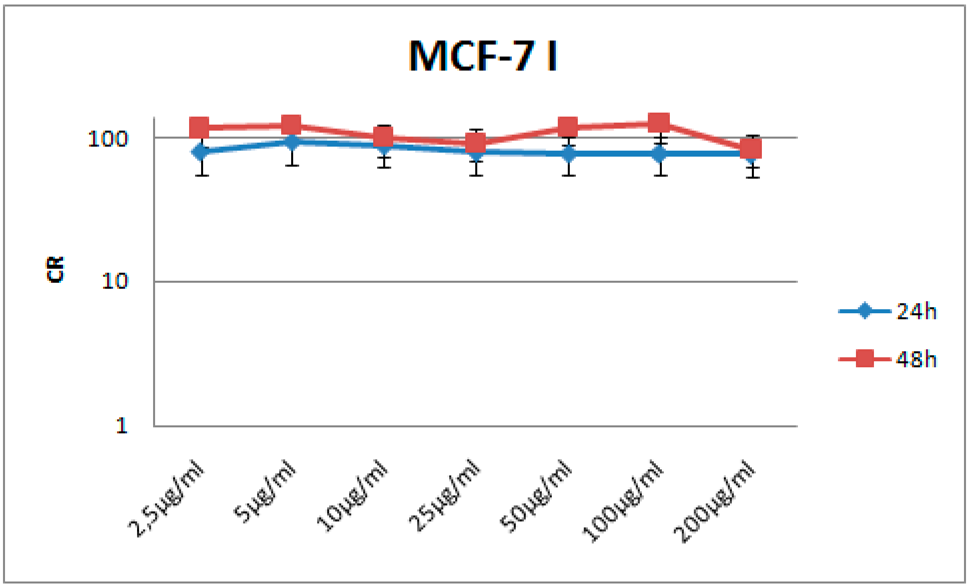

b

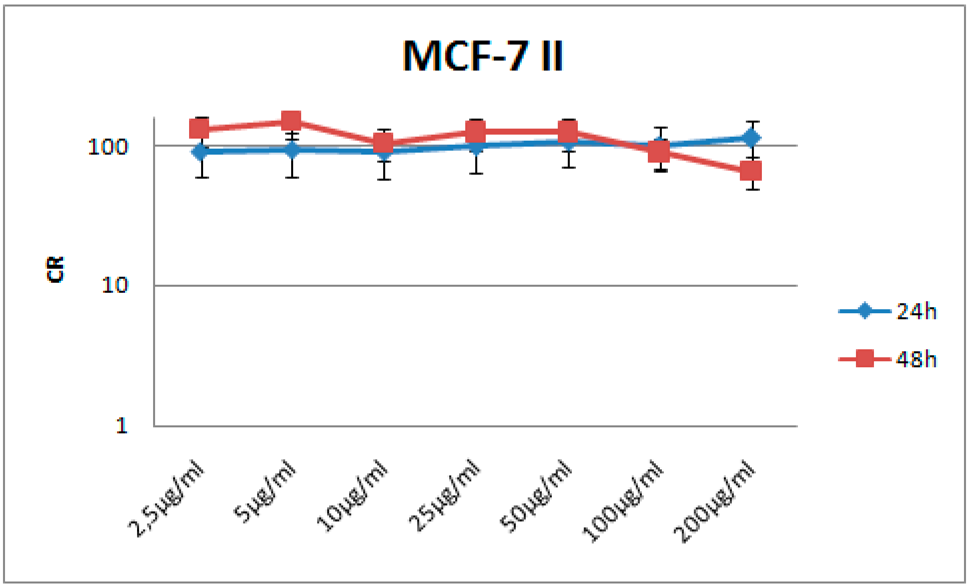

Fig S2. Cell viability (CR) related to breast cancer cells, MCF-7, after the treatment with two sponge sub-fractions named 1 (a) and 2 (b)for 24 and 48 h.

Fig S3

a

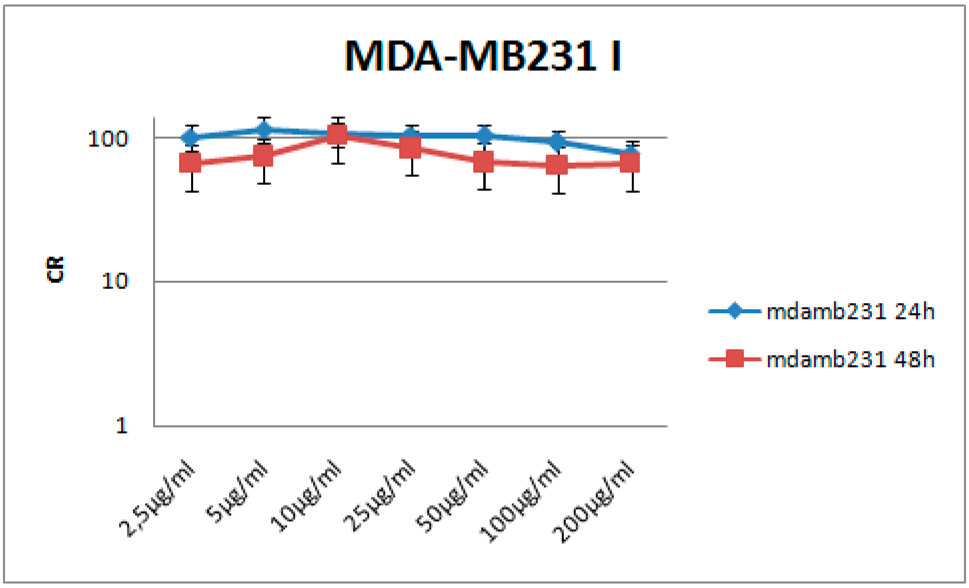

b

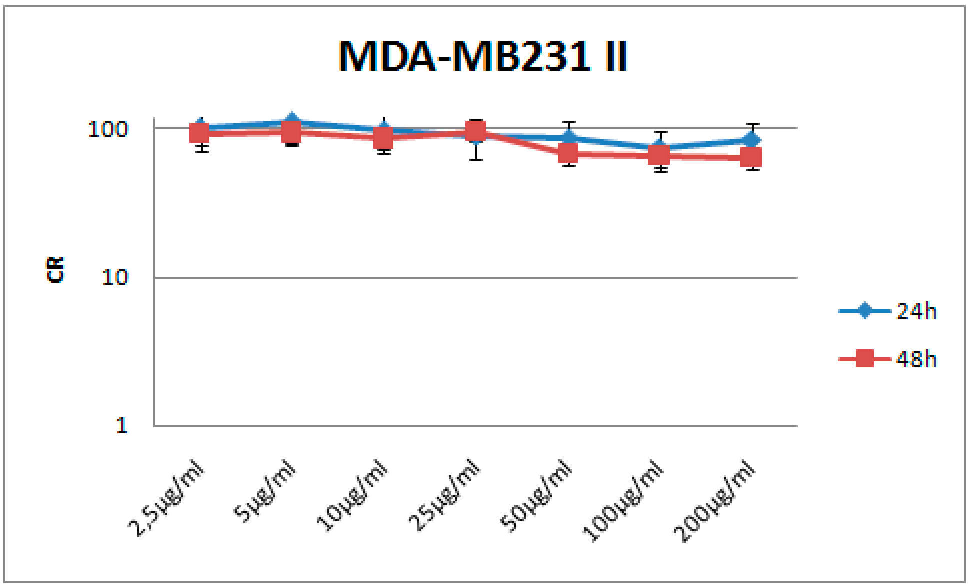

Fig S3. Cell viability (CR) related to breast cancer cells, MDA-MB231, after the treatment with two sponge sub-fractions named 1 (a) and 2 (b) for 24 and 48 h.

Fig S4

a

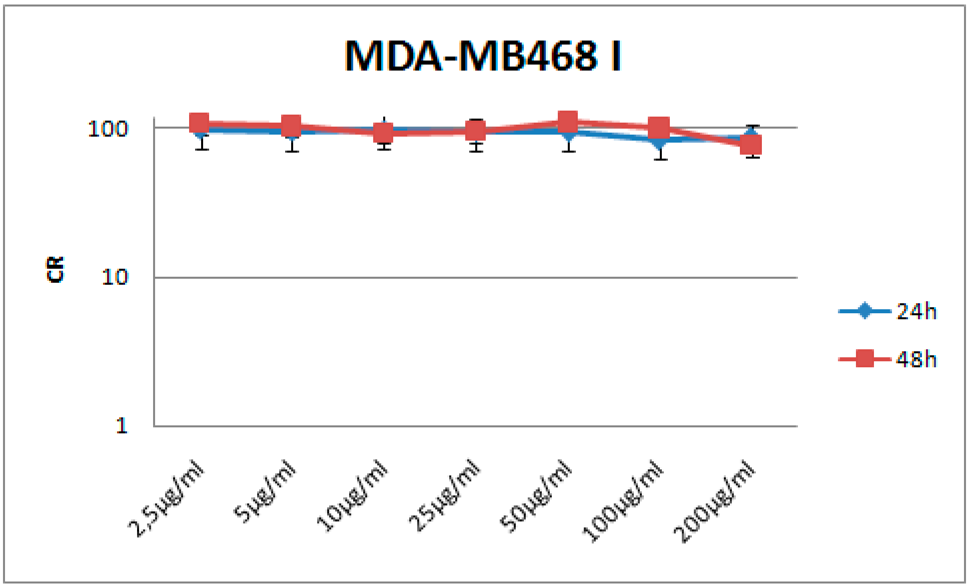

b

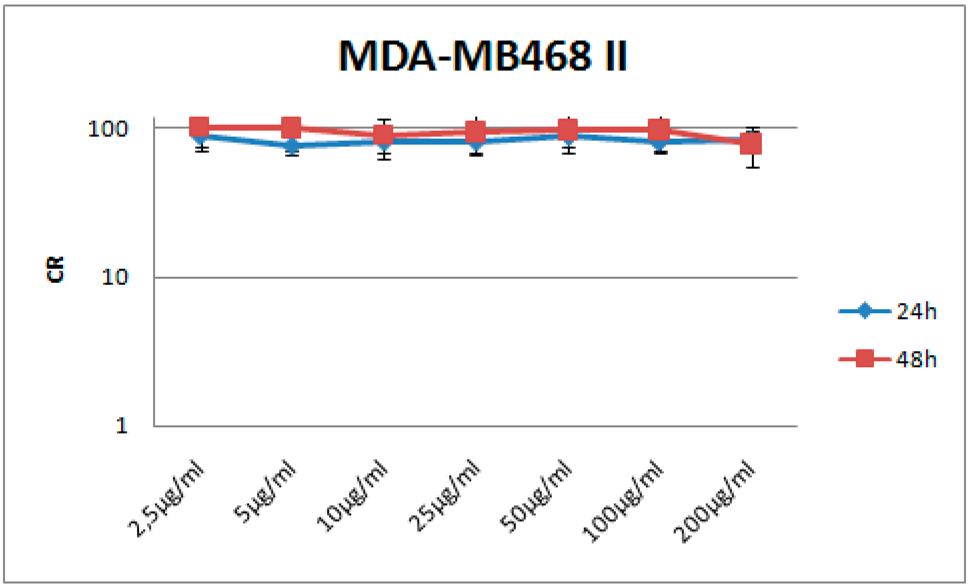

Fig S4. Cell viability (CR) related to breast cancer cells, MDA-MB468, after the treatment with two sponge sub-fractions named 1 (a) and 2 (b) for 24 and 48 h
